# *Stenotrophomonas maltophilia* endophthalmitis following keratoplasty

**DOI:** 10.1186/s12348-023-00368-9

**Published:** 2023-10-13

**Authors:** Bilge Tarım, Mücella Arıkan Yorgun, Birsen Özdem, Emine Kalkan Akçay

**Affiliations:** 1 Department of Ophthalmology, University of Health Sciences, Ankara Bilkent City Hospital, Ankara, Turkey; 2 Department of Ophthalmology, Yıldırım Beyazıt University, Ankara Bilkent City Hospital, Ankara, Turkey; 3 Department of Microbiology, University of Health Sciences, Ankara Bilkent City Hospital, Ankara, Turkey

**Keywords:** Endophthalmitis, Keratoplasty, *Stenotrophomonas maltophilia*, Descemetocele, Hypopyon

## Abstract

Endophthalmitis is among the most sight-threatening infections in ophthalmology practice. Many microorganisms causing endophthalmitis have been reported. *Stenotrophomonas maltophilia* is among the rare causes of endophthalmitis and has been reported after cataract surgery, intravitreal injections and ocular trauma. We report a case of *S. maltophilia* endophthalmitis after keratoplasty, which is a rare entity, in a 63-year-old female patient.

## Introduction

*Stenotrophomonas maltophilia* is a gram-negative bacillus which is opportunistic, nonfermenting, obligate aerobic and motile [1]. The microorganism is usually isolated from water, soil and plants [2, 3]. *S. maltophilia* infections are known as nosocomial infections due to their ability to live on plastic and glass surfaces [4]. Pneumonia, acute exacerbations of chronic obstructive pulmonary diseases, bacteremia, septicemia, cellulitis, myositis, osteomyelitis, meningitidis, endocarditis, urinary tract infections and biliary sepsis are *S. maltophilia* associated infections [5]. The most important ophthalmological diseases caused by *S. maltophilia* are keratitis, scleritis, conjunctivitis, preseptal cellulitis, dacryocystitis and endophthalmitis [6–10]. *S. maltophilia* is a rare cause of endophthalmitis and cases of endophthalmitis associated with *S. maltophilia* have generally been reported after cataract surgery, trauma or intravitreal injections [11–13].

When we searched the literature, we found very few cases of *S. maltophilia* endophthalmitis after keratoplasty. In this case, we present a patient who developed *S. maltophilia* endophthalmitis after a keratoplasty procedure.

## Case report

**Fig. 1:**
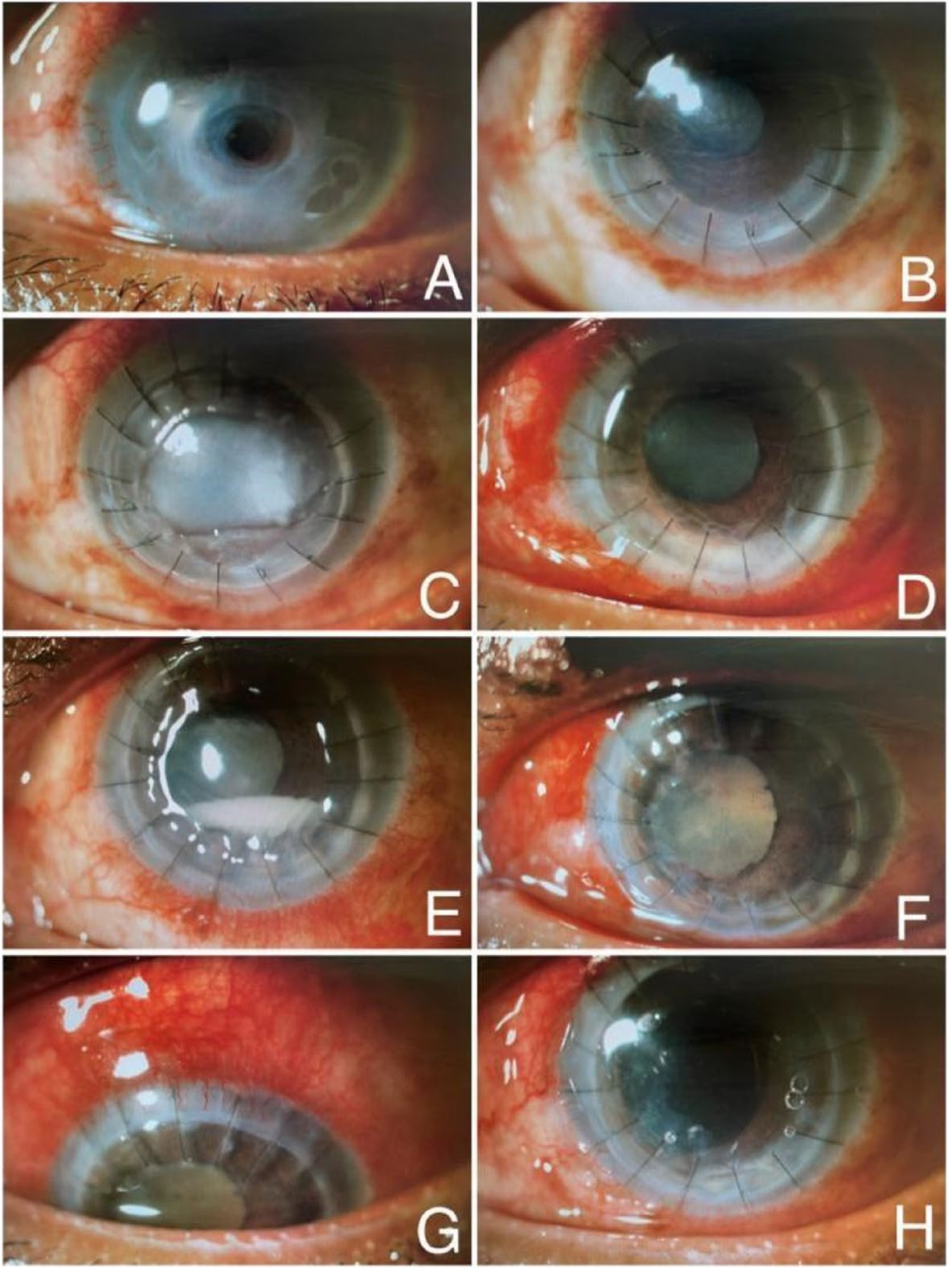
**A** Corneal central thinning and descemetocele at the time of first admission, **B** Anterior segment photograph after deep lamellar keratoplasty, **C** Graft failure due to endotheliitis, **D** Anterior segment photograph after penetrating keratoplasty, **E**–**F**-**G** Hypopyon in the anterior chamber, fibrinous reaction, mature cataract and ciliary injection, **H** Anterior segment photograph after phaco-vitrectomy

A 63-year-old female patient was referred to our clinic due to corneal thinning and melting in the left eye. Her best corrected visual acuity (BCVA) was finger counting from 20 cm in the right eye and finger counting from 10 cm in the left eye. Slit-lamp biomicroscopic examination revealed an opacity of 8 mm in the left cornea, thinning, and a central descemetocele (Fig. 1-A); while the right eye was pseudophakic, and penetrating keratoplasty (PKP) was previously performed on the right eye. She had optic atrophy on the right, and her history could only reveal that she had been treated for glaucoma for a long time. At the same time, the left eye was phakic, fundus examination could not be performed in detail due to corneal opacity, but no vitreous problems were observed while the retina was attached in the B-scan ultrasonography.

**Fig. 2:**
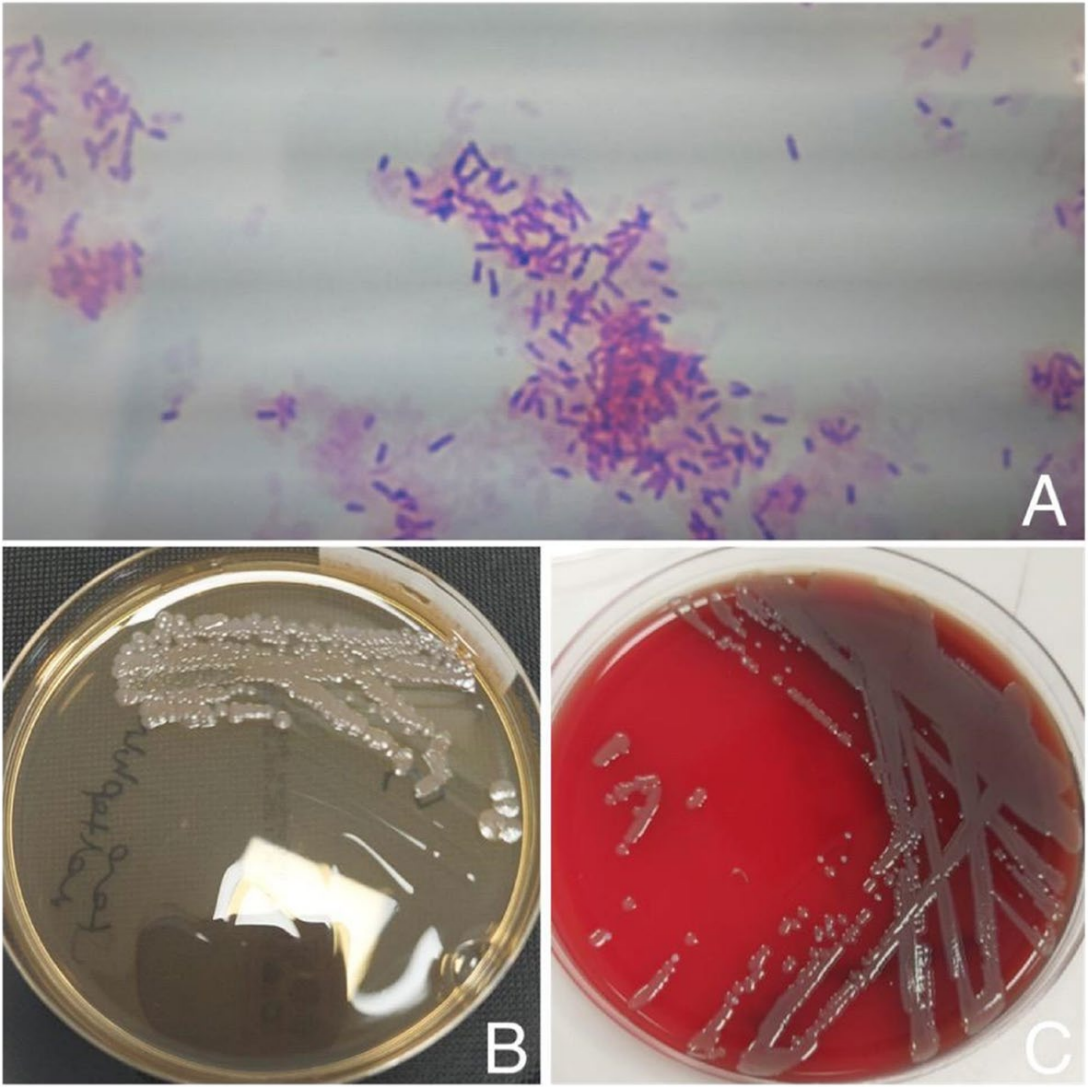
**A** Gram-negative bacillus S. maltophilia growth on hematoxylin and eosin (H-E) staining in the microscopic examination of vitreous and anterior chamber sample, **B** S. maltophilia growth on MacConkey agar, **C** S. maltophilia growth on blood agar

After deep anterior lamellar keratoplasty (DALK) was performed in the left eye, endotheliitis and endothelial insufficiency developed in the follow-ups, and PKP was applied to the left eye 2 weeks later (Fig. 1-B,C,D). Fibrin reaction started in the anterior chamber on the second postoperative day of the patient and it was observed that this reaction gradually increased. Subconjunctival dexamethasone was added twice a day to the patient who received topical dexamethasone 8 times a day, topical moxifloxacin 4 times a day, valacyclovir 1000 mg orally 3 times a day and acetazolamide 250 mg orally 3 times a day. The gradually increasing reaction was replaced by a 1.5 mm hypopyon in the first week, and additionally, posterior synechiae and vitreous condensation were found on B-scan ultrasonography (Fig. 1-E,F,G). With the preliminary diagnosis of endophthalmitis, samples were taken from the anterior chamber and vitreous, then intravitreal injections vancomycin (1 mg / 0.1 mL) and ceftazidime (2.25 mg / 0.1 mL) were given; and cefuroxime (1 mg / 0.1 mL) was administered to the anterior chamber. Topical fortified vancomycin and ceftazidime drops hourly and cyclopentolate 3 times daily were started. It was reported that *S. maltophilia* isolated in vitreous and anterior chamber samples (Fig. 2-A,B,C). According to the patient's antibiogram sensitivity result, intravenous levofloxacin 750 mg once a day was added to the treatment. Instead of topical fortified vancomycin, levofloxacin drops were started hourly. In the follow-ups, the hypopyon regressed and completely disappeared after 11 days. However, due to the development of phacomorphic glaucoma, narrowing of the anterior chamber in the left eye, and non-resolving vitreous haze on ultrasonography, combined phacoemulsification and pars plana vitrectomy were performed (Fig. 1-H). In her follow-ups, her cornea was clear, there was no reaction in the anterior chamber, no hypopyon was observed, and there was no vitritis.

## Discussion

*S. maltophilia* is an opportunistic infection that is a rare cause of endophthalmitis and cases of *S. maltophilia*-related endophthalmitis have often been reported following cataract surgery, trauma or intravitreal injections [11–13]. We present a case of *S. maltophilia* endophthalmitis after keratoplasty, which is a rare entity in the literature.

There are some cases in the literature who underwent intravitreal injection for various reasons and subsequently developed *S. maltophilia* endophthalmitis. Boeke et al. reported *S. maltophilia* endophthalmitis developing 1 month later in a 70-year-old female patient who underwent intravitreal aflibercept for diabetic macular edema. Due to the suspicion of endophthalmitis a tap procedure was performed and then intravitreal vancomycin, ceftazidime and dexamethasone were injected. Topical prednisolone acetate 1% every hour, topical moxifloxacin 4 times daily and cyclopentolate 3 times daily were started. Then *S. maltophilia* growth was observed in the patient's aqueous humor, and his clinical improvement was observed with the continuation of the topical treatment and the need for vitrectomy did not arise [13]. Our patient did not have severe pain as stated in this case. Although hypopyon was also observed in our patient, a fibrinous reaction occurred in the anterior chamber first, and then it was gradually replaced by hypopyon. Since condensation in the vitreous and phacomorphic glaucoma developed in the follow-up of our patient, combined phaco-vitrectomy surgery was performed.

Karakurt et al. reported 6 cases who developed *S. maltophilia* endophthalmitis between 1 and 19 days after cataract surgery [14]. In addition, Chang et al. reported 8 cases of *S. maltophilia* endophthalmitis that occurred following cataract surgery [11]. Vitrectomy was required in 3 of these 8 patients. Similarly, Ji et al. published 14 cases of *S. maltophilia* endophthalmitis that occurred between 1 and 56 days postoperatively after cataract surgery [15]. As can be seen, *S. maltophilia* endophthalmitis cases in the literature were generally seen after cataract surgery. In our patient, we encountered an endophthalmitis that gradually appeared after keratoplasty.

In addition to all these, there are also cases of *S. maltophilia* endophthalmitis reported after ocular traumas. Lai et al. reported a case of *S. maltophilia* endophthalmitis after penetrating injury by a wooden splinter [12]. Patton et al. brought to the literature a case of *S. maltophilia* endophthalmitis in a patient with intraocular metallic foreign body after trauma [16]. Also, Kherani et al. published a *S. maltophilia* endophthalmitis case following penetrating corneal injury [17]. As it is known, due to impaired sterility in intraocular penetrating injuries, we frequently encounter endophthalmitis as in these cases. However, we usually see rapidly progressive endophthalmitis in these patients. In our patient, the fibrin reaction that occurred after keratoplasty was followed for a while, then it was replaced by a hypopyon, but the size of this hypopyon remained more stable than in classical endophthalmitis.

In a case published by Díez-Álvarez et al., a case of *S. maltophilia*-associated keratitis-endophthalmitis has been reported. In this case, an 84-year-old female patient developed a persistent epithelial defect and a dense stromal infiltrate after descemet stripping automated endothelial keratoplasty (DSAEK) surgery and *S. maltophilia* growth has been reported in the corneal scraping sample taken. Complete recovery was achieved in 3 weeks after oral and topical trimethoprim-sulfamethoxazole (TMP/SMX) treatment. This case emphasized the importance of keeping in mind that *S. maltophilia* may also be a factor in keratitis after corneal transplantation [18]. In our case, there was a patient who developed *S. maltophilia*-related endophthalmitis after penetrating keratoplasty, presented with progressively increasing inflammation, fibrin reaction and vitreous condensation, and was more resistant to heal.

There is one case report describing *S. maltophilia* keratitis that developed after penetrating keratoplasty surgery [19]. In this case, a 70-year-old patient complained of decreased vision 5.5 months after the surgery and *S. maltophilia* was isolated in corneal scraping samples. In this patient, as in our patient, the treatment plan was shaped according to the antibiotic susceptibility test and it was observed that the keratitis was completely resolved in 2.5 months with topical 0.3% ciprofloxacin hydrochloride treatment. In our patient, unlike this patient, *S. maltophilia*-related endophthalmitis was observed after keratoplasty, and the time of occurrence in our case was seen earlier after keratoplasty.

## Conclusion

Although *S. maltophilia* is a rare cause of endophthalmitis, microbiological samples should be taken from the vitreous and anterior chamber when there are findings in favor of endophthalmitis on examination. We should start the appropriate endophthalmitis treatment without waiting for the culture result and adjust the treatment by switching to appropriate sensitive antibiotics when *S. maltophilia* grows. It can be confused with inflammatory conditions, such as anterior chamber fibrinous reaction. *S. maltophilia* should be kept in mind in endophthalmitis that develops after any interventional procedure to the eye.

## Data Availability

All data generated or analyzed during this study are included in this published article.
